# First 500 Fractions Delivered with a Magnetic Resonance-guided Radiotherapy System: Initial Experience

**DOI:** 10.7759/cureus.6457

**Published:** 2019-12-24

**Authors:** Bilgehan Sahin, Teuta Zoto Mustafayev, Gorkem Gungor, Gokhan Aydin, Bulent Yapici, Banu Atalar, Enis Ozyar

**Affiliations:** 1 Radiation Oncology, Acibadem Maslak Hospital, Istanbul, TUR; 2 Radiation Oncology, Acibadem University School of Medicine, Istanbul, TUR; 3 Radiation Oncology, Acibadem University School of Medicine, Acibadem Maslak Hospital, Istanbul, TUR

**Keywords:** magnetic resonance guided radiotherapy, mr-linac, online adaptive radiotherapy, cine mri, magnetic resonance-guided radiation therapy (mrgrt), sbrt

## Abstract

Objectives

Improved soft-tissue visualization, afforded by magnetic resonance imaging integrated into a radiation therapy linear accelerator-based radiation delivery system (MR-linac) promises improved image-guidance. The availability of MR-imaging can facilitate on-table adaptive radiation planning and enable real-time intra-fraction imaging with beam gating without additional exposure to radiation. However, the novel use of magnetic resonance-guided radiation therapy (MRgRT) in the field of radiation oncology also potentially poses challenges for routine clinical implementation. Herein the early experience of a single institution, implementing the first MRgRT system in the country is reported. We aim to describe the workflow and to characterize the clinical utility and feasibility of routine use of an MR-linac system.

Methods

The ViewRay MRIdian MR-linac system consists of a split-magnet 0.35 T MR-imaging scanner with a double focused multi-leaf collimator (MLC) equipped 6MV linear accelerator. Unique to the system are the control console integrated on-table adaptive radiation therapy (oART) planning capabilities as well as automated beam gating based on real-time intra-fraction MR imaging. From the first day of clinical implementation, oART was performed according to physicians’ discretion when medically indicated. All fractions were delivered under real-time imaging with soft tissue-based automated beam gating with individualized gating boundary settings. Patients actively assisted in breath-hold beam gating with the help of custom designed prismatic glasses allowing sight of a computer monitor mounted on the back wall just behind the MRI system bore. Patient demographics and treatment experience, indications for MRgRT including diagnosis and disease site, radiation dose prescribed and fractionation scheme, utilization of oART, respiratory gating settings, as well as duration of each treatment phase were analyzed.

Results

Between September 2018 and May 2019, 72 patients with 84 tumor sites were treated with MRgRT in 500 total fractions. Median patient age was 66 years (range: 28-83 years). Among 84 tumor sites, the most frequently treated regions were upper abdominal and pelvic (n = 36, 43% and n = 29, 34%, respectively). The most common diagnosis was prostate cancer, with 14 patients treated. In 69 patients (93.2%) oART was used at least once during a treatment course. Twenty-nine targets (43.1%) with significant breathing-related motion were treated in breath-hold with patient visual feedback. Median prescribed dose was 36.25 Gy (range: 24-70 Gy) in median five fractions (range: 3-28 fractions). A gating boundary of 3 mm around a gating region of interest (gROI) was most commonly used (range: 3-5 mm) with 95% of the gROI (range: 93-97%) required to be within the gating boundary for the beam to automatically engage. Mean total treatment time was 47 min (range: 21-125 min) and mean beam-on time was 16.7 min (range: 6-62 min).

Conclusions

MRgRT afforded by an MR-linac system has been successfully implemented into routine clinical use at our institution as the first system of its kind in Turkey. While the overall number of patients treated and fractions delivered is still limited, we have demonstrated the feasibility of both on-table adaptive radiation therapy as well as automated real-time beam gating on a daily basis in acceptable time schedules.

## Introduction

With the promise of better soft-tissue visualization, the use of magnetic resonance imaging (MRI) in radiation oncology is vastly increasing. While historically diagnostic scans were used to derive additional imaging information for radiation therapy (RT) planning, today, a number of institutions have installed dedicated MR-simulators. The integration of a MR-imaging system with a RT delivery device has transferred the ability to acquire MR-images with the patient in RT treatment position into the treatment vault. The availability of high-quality MR-imaging has been demonstrated to facilitate the process of online image-guided radiation therapy and can also enable the medical decision making if RT plan adaptation may be required during a course of RT. Equally compelling is the ability to continuously image the targeted region while the radiation dose is being delivered, without additional radiation exposure.

However, technical challenges to integrate an MR-imaging system into a radiation delivery device had initially limited beam delivery to isotope-based radiation sources, specifically Cobalt 60 (MRIdian, ViewRay Inc, Oakwood Village, OH). With this integration, radiofrequency noise, produced by linear accelerator-based radiation sources that would have significant, detrimental impact on MR-image quality, was avoided. Only more recently have these challenges been solved and today multiple linear accelerator-based MR-guided radiation therapy systems have become commercially available. While the available systems differ in their concept and degree of integration of the linear accelerators into the imaging system, they both promise to advance the capabilities to address tumors in locations difficult to image using on-board cone-beam CT or megavoltage CT systems.

Conventional image-guided radiotherapy (IGRT) techniques, such as KV, MV or Cone-Beam CT, uses undesirable radiation exposure [1]. Besides the ability of the MR-linac to avoid this undesirable radiation exposure, it can also monitor intra-fraction motion by safely visualizing the patient’s whole treatment fraction with Cine MRI (cMRI) mode. Moreover, MR-guided IGRT imaging can also visualize daily inter-fractional changes in anatomy and permit faster plan adjustments, especially in abdomen and pelvic soft tissue regions which can hardly be seen on conventional X-ray-based IGRT. These daily/on table adjustments on main plan enable superior sparing of organs-at-risk (OAR) and safer dose escalation on the tumor [2]. Whole of these on-table efforts increasing therapeutic ratio of RT are termed as online adaptive radiation therapy (oART). With the dedicated treatment planning system, MR-guided IGRT images can be easily utilized and new daily anatomy adapted plans can be generated routinely [3,4].

Early reports have demonstrated the successful implementation of these systems into a number of clinics world-wide. Today, also a number of retrospective outcome analyses and a smaller number of phase one and phase two prospective clinical trials suggest the clinical value of investing in this technology [5-7].

In this report, we summarize our early experience of integrating an MR-linac system, MRIdian Linac (ViewRay Inc. Oakwood Village, OH), into clinical routine at a private hospital in Istanbul (anonymized for review), the first installation of its kind in Turkey. Based on an analysis of the first 500 fractions delivered, we aim to describe patient selection criteria, the clinical workflow and treatment options afforded by the system that differ from the technical abilities of other radiation delivery systems in our institution.

## Materials and methods

The MRIdian Linac system consists of a split, super-conductor low-field (0.35 tesla) MRI system with a 70-cm bore and a 50-cm field-of-view (FOV). The associated radiation delivery system consists of a ring-gantry mounted 6 MV linear accelerator and double-focused multi-leaf collimator (MLC). With a source axis distance of 90 cm, the flattening filter-free linear accelerator has a 6 Gy/min dose rate at isocenter with a 27.1 x 24.5 cm maximum field size. The integrated treatment couch has 3 degrees of freedom to accelerate set-up process without having to reposition the patient.

A dedicated Monte Carlo-based treatment planning system is delivered with the system and is also integrated into the treatment console to enable on-table adaptive re-planning should this be considered medically necessary.

Volumetric MR imaging is provided for image-guidance and assessment of changes in target volume or the relationship between target volume(s) and organs at risk. Single or multi-slice real-time imaging at up to 4 frames/second allows to visualize the target and nearby organs at risk during dose delivery with simultaneous algorithm-based soft-tissue tracking and automated beam control (beam gating).

General considerations for use of an MR-linac

Due to the strong magnetic field associated with the MRI system, no ferromagnetic instruments or devices are allowed in the treatment room. For example, patient transportation into the treatment room, if needed, is done by an MRI compatible stretcher or wheelchair.

MR safety training was administered to all staff including physicians, medical physicists, technicians as well as cleaning and maintenance personnel. Specific consideration needs to be given to patient selection. While implanted devices such as screws and plates or joint replacement hardware is often compatible with MR imaging, additional precautions need to be directed towards establishing MR compliance of other devices such as pacemakers and other medical implants. While tattoos and permanent make-up do not constitute a general contraindication for MR-imaging, their presence is always documented in the patient’s medical chart. Loose metals inside a patient’s body, such as metal flakes in an eye, or other metals inside a patient’s body (such as shrapnel) constitute contraindications for placement into an MR-imaging system and consequently into an MR-linac.

From a radiation oncology perspective, all patient positioning must be done with MR compatible immobilization equipment. Equally, all dosimetry equipment must be MRI compatible, in our case requiring acquisition of dedicated dosimetry equipment.

Simulation and treatment planning

As an all-in-one simulation, dose planning and treatment delivery system, the MRIdian Linac differs from conventional CT-based radiation delivery systems in several aspects:

Patient Positioning/Setup

**Figure 1:**
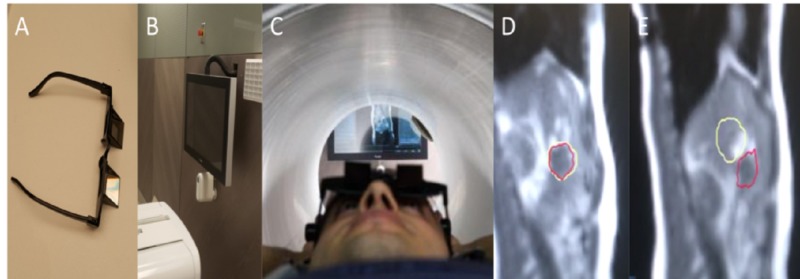
Equipment, patient set-up and cine-MR (A) Prismatic glasses, (B) rear monitor showing cine-MR, (C) patient set-up, (D) breath hold position target (yellow-line) and tumor (red-line) are overlapping which allows irradiation. (E) Rest position, target (yellow-line) and the tumor (red-line) separated each other and system automatically stops irradiating.

Firstly, body coils are essential for MR scanning; headphones and a call button can also be used. To minimize the respiratory movements, according to physicians’ discretion which is affected by tumor site and patients` performance, prismatic glasses can be utilized. These glasses allow 90-degree bending of patients’ vision enabling them to see the screen just posterior to the patient and the linac (Figure 1).

Low Resolution Scan

This is a short scan to see and correct the position of the patient. It generally takes less than 20 seconds.

High Resolution Scan

This is the high-resolution scan to detect inter-fractional daily anatomical changes of the tumor site and OAR for further contouring and adaptive planning. Based on the size and resolution of the area, the duration of the scan may vary from seconds to 12 min. In order to enhance discriminative power of the MRI, MRI contrast agents such as iopamiron, gadoxetate (Gadovist, Primovist) can be used. At our institution, especially for abdominal tumors we try to simulate patients with an empty stomach and then contrast agents can be used if the target cannot be adequately distinguished.

Contouring

Deformable registration for the surrounding tissue and minor adjustments of gross tumor volume (GTV) are initially made. Air-water contouring is added manually for electron density adjustments. GTV and OAR contours are finalized for planning and a new DVH is generated in order to compare to known dose constraints.

Plan Prediction

Re-planning & plan comparison - QA: After contouring the minor adjustments for daily anatomy, the main plan is framed according to last contour with daily anatomy and a predicted plan with daily anatomy is reconstructed. Dose constraints and the DVH are compared with original unchanged plan. Dose changes with daily anatomy can clearly be seen in this feature. If any of the GTV - planned target volume (PTV) or OAR dose is violated, the main plan is swiftly re-optimized for violated constraints. Re-optimized plan is revised for violation of the constraints and can be compared with either original or predicted plan. If this plan is not satisfactory, a new plan can be performed by adjusting fields and monitor units of each beamlets once more. After plan is accepted, a fast-online quality assessment would be done sequently.

Treatment Delivery

After the plan is finalized, treatment is started. On-time cMRI enables 2D-tracking on sagittal plane. A sagittal plane which includes tumor is chosen, especially we prefer a plane which includes contrast differences the most in order to ensure tumor recognition and tracking. According to patient performance and treatment site, respiratory gating can be utilized. For gating, we utilize prismatic glasses which enable patient to see the screen on the back, displaying patient’s own respiratory movements (Figure 1). On the screen, patient can see real-time cMRI. In this scene, yellow line refers to irradiated area and the red line (automatically detected by software's algorithm) refers to target/tumor site and the patient actively adjusts his/her respiratory movements to ensure the irradiated site and the tumor site are overlapping. The ratio of the intersection of yellow and the red lines is calculated by the system. The ratio of the tumor area outside the irradiate area is called the Range of Interest (ROI) value and can be assigned as a cut-off value for the system to automatically stop the treatment. In our institution, we try to keep this ratio under 7% (Video 1).

**Video 1:** Cine MR: target and tumor positioning

## Results

Since September 2018, 72 patients with 84 tumor sites were treated with MRIgRT in 500 total fractions. The median age was 66 years (range: 28-83) and 69% were male. The most commonly treated regions were upper abdominal (n = 36, 43%) and pelvic regions (n = 29, 34%). The most commonly treated diagnoses were prostate cancer (n = 24, 33%) and lung cancer (n = 15, 21%).

RT indications included definitive (n = 33, 46%) oligometastatic (<3 metastasis) (n = 34, 46%) palliative, preoperative and re-irradiation. The vast majority of patients (n = 65, 90.2%) were treated with stereotactic body radiosurgery (SBRT-ART was a part of the work-flow and was used routinely). In only five patients (6.8%) ART was not utilized.

Respiratory gating with cMRI was used from the beginning with MR-linac. Decision to use respiratory gating is made according to target site and tumoral displacement by respiratory movement. In prostate cancers gating is not used routinely. Thirty-five patients (49%) were treated without respiratory gating, whereas 37 patients (51%) were irradiated using the breath-hold technique. In early results, breath-hold technique shortened total treatment duration (mean: 49.2-44.5 min) by shortening treatment application time, imaging time, plan re-optimisation time.

The median dose was 36.25 Gy (range: 24-70 Gy) in a median of 5 fractions (range: 3-28). The most commonly used boundary was 3 mm (range: 3-5 mm) and treatments often applied a limit of 5% ROI (range: 3-7%). The mean total treatment time was 47 min (range: 21-125 min) and mean beam-on time was 16.7 min (range: 8-76 min). During 105 (21%) fractions treatment was interrupted due to patient movements. Only one (0.2%) fraction was delayed due to technical difficulties with the system. Treatments were well tolerated and none of the patients stopped treatment due to discomfort or intolerability.

## Discussion

The MR-linac system has many advantages including better soft-tissue visualization, better real-time IGRT with cMRI and fast planning enabling on-table re-contouring and re-planning, the so-called ART. However, it has some difficulties such as commissioning the system and implementing a different workflow [2-4]. Commissioning and hardware incompatibilities are not the topic of this report and will be discussed in another paper. The work-flow differences are handled differently in each center but mainly grouped in two approaches. The first strategy is starting with the similar conventional work-scheme and adding MR-linac specific features one by one. For example, starting with simple 3-dimentional conformal palliative plans initially and after that gradually adding definitive IMRT planning, radiosurgery planning, IGRT with cMRI, respiratory gating techniques and ART, also slowly allocating limited institutional human-power to this area. This approach is found to be safer to adapt, but it needs longer time frame to utilize all the new features and potential benefits of the MR-linac system. It also demands more time to recuperate the setup cost of the system which can be a major problem in private hospitals or institutions.

The second approach is diverging all available sources to the area and initiating all features of the system immediately from the first day of the treatment and trying to gain faster adaptation to the new work-flow. In our institution, whole features were utilized all together with the initiation of the system in order to adapt faster to novel work-scheme. We believe, with dedicated trained human resources full-speed adaptation from the first day is feasible.

We believe the MR-linac system is utilized most cost-effectively by radiosurgery. While having slightly longer total treatment durations, the number of fractions is greatly reduced and seems more cost-effective. Excluding seven patients (9.8%) who needed more fractions to ensure safer OAR dose limitations, all patients applied SBRT. We believe this is a high ratio among other centers using MR-linac. According to a previous publication by Washington University, they reported 94 patients with SBRT (30%), 76 patients (24%) with 3D conformal RT, and 146 patients (46%) with IMRT in 316 patient series [8]. Two years later they reported their updated results with 666 courses of MRIgRT including 266 SBRT (39.9%), 240 IMRT (36%) and 160 3D conformal RT plans [9]. In our institution, we mostly use MR-linac for radiosurgery purposes.

Upper abdomen and liver SBRT

**Figure 2:**
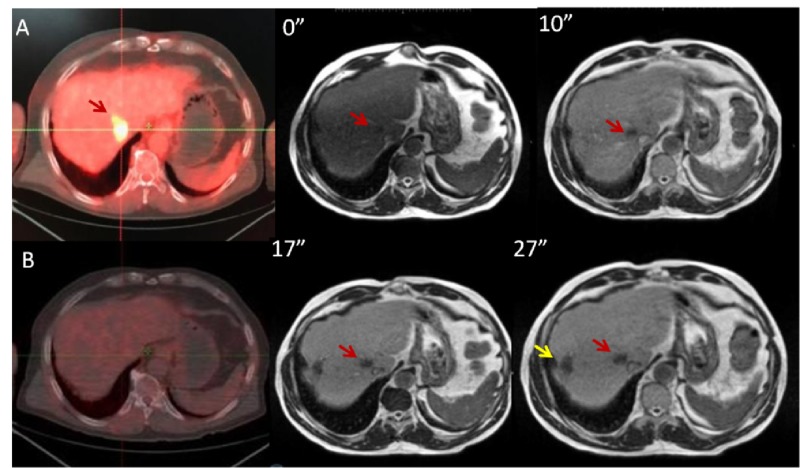
PET-CT and MRI scans with gadoxetate (A) PET-CT scan before treatment, (B) 3rd month follow-up PET-CT scan after radiosurgery, 0-10-17-27 minutes of gadoxetate injection: Red arrow denotes tumoral target, yellow arrow denotes previous treated area with interventional radiology.

With better soft-tissue visualization, accurate IGRT with cMRI and enabling ART to adapt daily anatomical changes of OAR like bowels and stomach, MR-linac can be widely used in areas where conventional systems cannot [10]. With conventional systems, abdominal region is the most feared region to perform radiosurgery. Even during conventional RT, it is difficult to escalate doses without potential OAR risks. The abdominal region is the most compatible region in which MR-linac features can be efficiently applied. For this reason, we mostly use MR-linac for abdominal SBRT with 36 regions (43%) treated in the upper abdomen. We use PetCT and MRI fusion to help in the generation of the main plan. We believe 0.35 T MRI is enough for ART and IGRT in most cases but in cases when target and OAR cannot be discriminated, a contrast agent gadolinium or gadoxetate disodium can be used. We prefer gadoxetate disodium for liver metastases for its ease to use and relatively lower side effect profile (Figure 2) [11]. Liver is positively enhanced by leaving tumoral site darker to improve recognition.

Prostate SBRT

**Figure 3:**
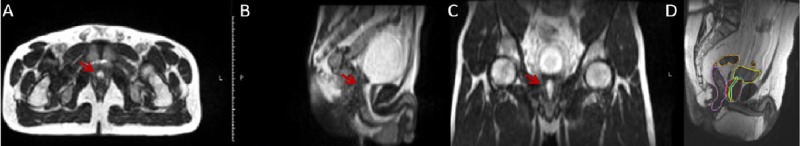
MRI scans showing urethra during micturation and contouring (A-C) Axial, sagittal and coronal simulation MRI scans of volunteer with red arrow showing urethra during micturation. (D) Sagittal view of simulation MRI scan and prostate, urethral and other OAR contouring.

Both robotic arm-based or linac-based fiducial tracking for prostate cancer radiosurgery is applicable in our institution. MR-linac-based radiosurgery is chosen in patients in whom fiducial placement is not preferred or contraindicated. Tracking without any intervention is a valid advantage to be chosen by patient and also physician. Another advantage is on-table adaptation and urethral sparing. Daily anatomical changes as bladder and rectum, change prostate position, so it is almost impossible to delineate urethral contour with Cone Beam CT (CBCT). Mostly a pediatric catheter was introduced for accurate urethra delineation; MR-linac gives a chance to delineate without having the comorbidity of a catheter. Firstly delineation exercises were made on volunteers’ MRI scans, and also to improve visualization of urethra, micturating urethrography MRI scans of volunteers were gained in setup position. A study showed MRI-guided delineation practices and displayed that urethra is not a tube-shaped structure but actually inverted ‘V’ or ‘Y’-shaped structure [12]. Our low-field MRI also could able to show structure as study describes (Figure 3). After re-contouring and ART, dose constraints adopted from Novalis Circle Phase II Trial (ClinicalTrials.gov Identifier NCT01764646) were used. Precise IGRT with cMRI is also crucial for performing successful treatments. Conventional CT-based systems do not have the ability to track all the intra-fractional movements of target and also OAR unless having a fiducial implemented. Since in radiosurgery both target and OAR dose is important, gathering and using all inter- or intra-fractional movement data for constructing internal target volume (ITV) and PTV is the safer way. A study used real-time MR-linac data to successfully predict tumor motion in thoracic and abdominal lesions [13]. In our institution, target and OAR motion data is continuously filed in order to maximize treatment precision of the radiosurgery. cMRI shows movements during entire treatment so unexpected bowel movements can be observed (Video 2).

**Video 2:** Cine-MR: Bowel movements

Future perspectives

**Figure 4:**
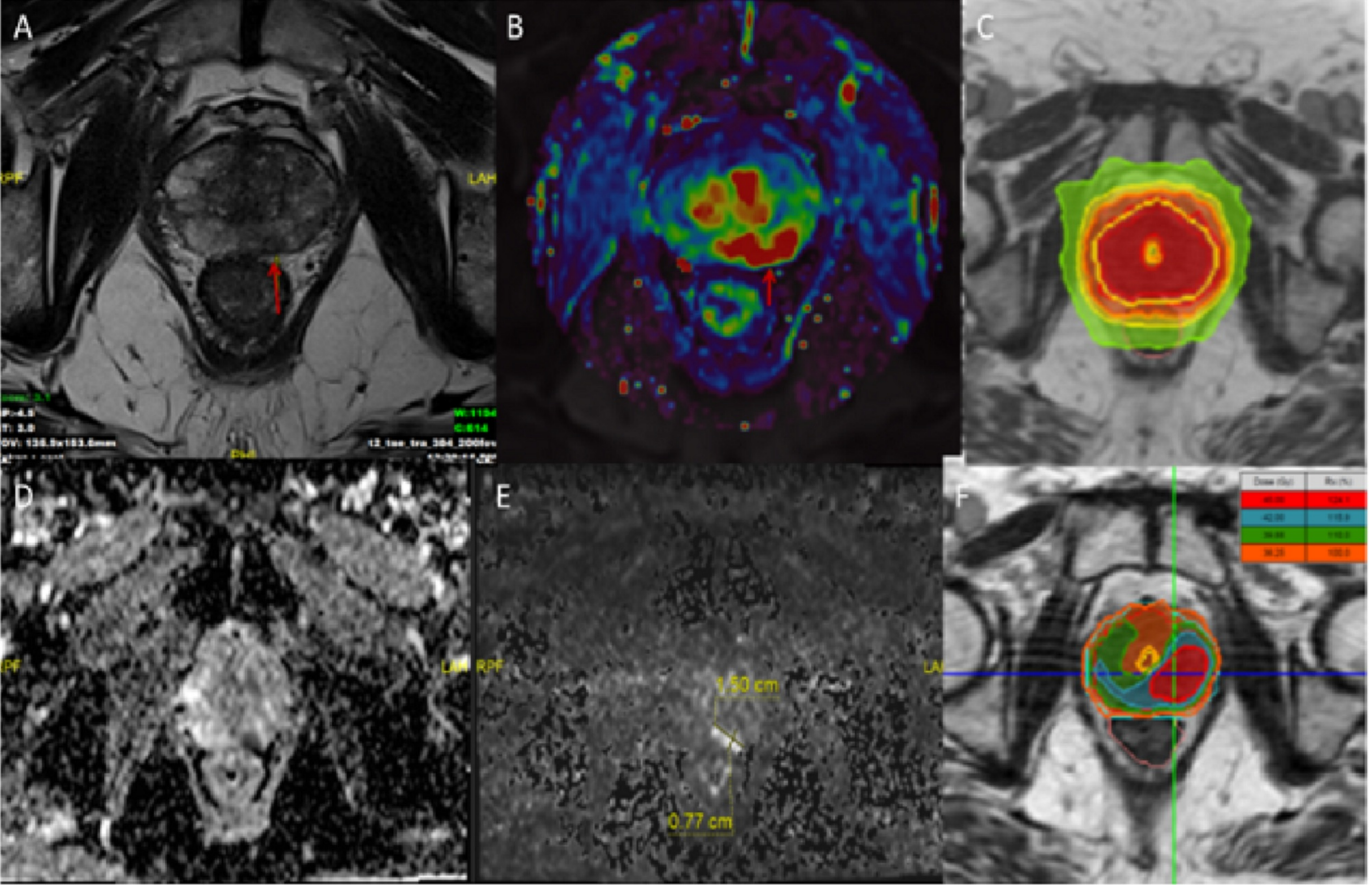
MRI scans and radiotherapy planning (A, B, D and E) Axial views of T2, K-trans, perfusion and diffusion MRI scans. (C) Homogeneous plan allowing homogeneous dose to whole prostate with urethral sparing. (F) Heterogeneous plan to escalate dose on resistant tumor segments.

Since the Harlow and Eisenbeis study in 1973, we realized imaging studies can provide additional information about tumor biology and nature [14]. Computer-aided diagnosis or radiomics is an emerging field which utilizes neural networks to artificially combine radiology, proteomics, genetics and all the information that novel technology brings. Radiomics can be applied to many tumor types and may have potential to change many tumor diagnosis and treatment guidelines. In radiotherapy, oART is the key part to adapt treatment to the changing tumor biology. Neural networks can be applied in either contouring tumor or auto-segmentation of the OAR. Today with oART, an MRIgRT system can adapt daily anatomical changes; in near future they can utilize daily biological changes. For instance, radiomics data may display radio-resistance segments in tumor volume and an oART plan can easily produce a heterogenous dose escalated hotspots and overlap these hotspots to these resistant segments with integrated boost techniques (Figure 4).

MR-linac system, despite the fact that it contains a 0.35 T low-field MRI, with the help of neural networks can also be used for prediction of disease prognosis. Boldrini et al. described in their 16 rectal cancer patient study that changes in radiomic features could predict complete response of rectal cancer treatment, even in the second week of treatment. The authors proposed it may add promising resources to current personalized medicine in rectal cancer care [15].

## Conclusions

Integration of a completely new paradigm in RT workflow (on-table recontouring, re-planning, online QA, real-time tumor tracking with MRI) can be challenging and intimidating at first. For this reason, centers who had been using MR-linac systems for longer periods of time had to proceed cautiously but steadily in order to implement and profit from the vast capabilities of the system. Evidence regarding safety, toxicity, efficacy of the new system is starting to grow exponentially. Collaboration between centers has made it possible for our team to achieve confidence and the ability to proceed with treatments with this MR-linac and its “ART” functionality in a relatively short period without any unexpected toxicities.
